# Evaluation of a Wearable Hydration Monitor for Estimating Sweat Loss and Hydration Status in Collegiate Athletes: A Pilot Study

**DOI:** 10.3390/nu18193166

**Published:** 2026-09-25

**Authors:** Jill McCann Snyder, Lindsay Toth, Elysse Aguero, Colleen Brennan, Kassandra Duarte

**Affiliations:** 1 Department of Nutrition and Dietetics, Brooks College of Health, University of North Florida, Jacksonville, FL 32224, USA; elysse.aguero@gmail.com (E.A.); n01487735@unf.edu (C.B.); n01635978@unf.edu (K.D.); 2 Department of Clinical and Applied Movement Sciences, Brooks College of Health, University of North Florida, Jacksonville, FL 32224, USA; l.toth@unf.edu

**Keywords:** sweat loss, hydration assessment, validity

## Abstract

Background/Objective: Instantaneous, accurate assessment of hydration and sweat loss has potential to optimize athlete performance and reduce risks associated with excessive exercise-related fluid loss. Reusable wearable hydration monitors, such as hDrop monitors, are an emerging non-invasive, quick, and easy to use alternative to existing methods. Purpose: To assess the accuracy of the hDrop wearable sweat monitors for estimating sweat loss and hydration status compared to established field-based measures, including body mass change and urine indices. Methods: Division I male collegiate soccer athletes contributed repeated training-session observations (38 total observations; 18 complete observations for sweat-loss analyses and 36 for hydration-score–USG analyses). They completed training sessions during which pre- and post-exercise body mass, fluid intake, urine output, and urine samples were collected. Sweat loss was calculated using changes in body mass adjusted for fluid balance. hDrop-derived estimates of sweat loss and hydration status were compared to criterion measures using mean bias, percent of criterion, mean absolute percent error (MAPE), repeated-measures Bland–Altman analysis, and generalized estimating equations accounting for observations clustered within athletes. Associations between hydration scores and urine-based markers were assessed using generalized estimating equations accounting for observations clustered within athletes. Results: The hDrop generated a sweat-loss estimate for 18 of 38 training-session observations (47.4%). Repeated-measures mean bias for sweat loss was −0.423 L (95% CI −1.106 to 0.259 L). The hDrop estimates averaged 106.2 ± 64.8% of criterion while MAPE was 50.3%, reflecting large individual error. In the repeated-measures analysis, criterion-measured sweat loss was positively associated with hDrop-estimated sweat loss (B = 0.204, 95% CI 0.018 to 0.391, *p* = 0.032). Significant proportional bias was present (slope = −0.790, 95% CI −1.438 to −0.143, *p* = 0.020), indicating greater underestimation at higher sweat-loss values. In repeated-measures GEE analyses, USG was not significantly associated with the hDrop hydration score either before practice (B = −407.9, 95% CI −1008.7 to 192.8, *p* = 0.183) or after practice (B = −135.0, 95% CI −803.8 to 533.7, *p* = 0.692). Conclusions: The hDrop generated sweat-loss estimates in fewer than half of training-session observations and demonstrated substantial individual-level error and significant proportional bias when estimates were available. Although the instantaneous hydration score decreased following exercise, its lack of association with USG leaves its physiological meaning and criterion validity uncertain. These findings do not support use of the hDrop as an interchangeable substitute for established methods of individualized sweat-loss or hydration assessment.

## 1. Introduction

Maintaining adequate hydration is important for athletic performance, recovery, and overall good health. During exercise, sweating plays a key role in thermoregulation by dissipating heat; however, excessive fluid loss, if not adequately replaced, can reduce total body water thereby increasing physiological strain [1,2]. This strain can include reduced plasma volume, increased cardiovascular demand due to higher heart rate and lower stroke volume, impaired thermoregulation, and earlier onset of fatigue during exercise or competition [3,4]. Fluid deficits may also negatively affect endurance performance, cognitive function, heat tolerance, and postexercise recovery, particularly when exercise is prolonged or performed in hot environments [4,5,6]. Because sweat losses vary according to exercise intensity and duration, environmental conditions, sport demands, and individual physiological characteristics, fluid-replacement needs can differ substantially among athletes [7]. Accordingly, practical methods for assessing hydration status and sweat loss may help athletes and sport professionals develop individualized hydration strategies.

Several methods are currently used to assess hydration status and exercise-related fluid loss. Commonly, due to its accessibility, changes in body mass measured before and after exercise are used to estimate whole-body sweat loss when fluid intake and urine losses are considered [8]. This approach may also be used to calculate sweat rate and inform individualized fluid-replacement recommendations. However, body-mass measurements can be influenced by recent food and fluid intake, urine and fecal losses, respiratory water loss, substrate utilization, metabolic water production, and the timing of measurement [8]. Hydration status can also be assessed using urine specific gravity, urine osmolality, or plasma osmolality. Urine specific gravity provides an estimate of urine concentration using a refractometer, whereas urine and plasma osmolality reflect the concentration of dissolved solutes in body fluids [8]. Although these methods provide useful information, they generally require discrete sampling, specialized equipment, or laboratory procedures and do not provide continuous feedback during training or competition. Their limitations also make them less accessible than assessing difference in body weight.

Advances in wearable technology have created opportunities for more accessible hydration monitoring in athletic, field-based, or free-living settings. Wearable sensors can collect physiological or biochemical information across a variety of exercise and activity types, in diverse settings, and may provide continuous, non-invasive, or minimally obtrusive feedback outside of laboratory environments [9,10]. Sweat is a particularly promising biofluid for wearable monitoring because it can be collected during exercise and contains electrolytes, metabolites, proteins, and hormones that may provide physiologically meaningful information [11,12]. Wearable hydration sensors use a range of electrical, optical, and physiological sensing approaches to estimate hydration-related outcomes [10,13,14]. However, variability in sweat composition, sweat rate, environmental conditions, and the relatively low concentration of some biomarkers may affect measurement accuracy [11,15,16].

Despite the growth of wearable health-monitoring technologies, there is a paucity of independent validation studies of commercially available hydration wearables in athletic populations [14,17]. Existing validation studies have often been conducted under controlled laboratory conditions and with relatively small samples, which may limit their applicability to real-world training environments [17]. Field-based validation is therefore needed to determine whether wearable hydration monitors provide sufficiently accurate estimates when used during athletic practice and competition. For devices intended to support individualized hydration decisions, evaluation of validity should consider not only average agreement at the group level but also the magnitude and consistency of error for individual measurements. Because this was an exploratory pilot study, no a priori threshold for acceptable measurement error was specified.

The hDrop Gen 2 is a reusable, sweat-based wearable sensor designed for athletic use. The device is marketed as providing real-time estimates of hydration status, sweat loss, and electrolyte loss through a connected mobile application [17,18]. Because the hDrop obtains a localized sweat-related signal from the upper arm, regional differences in sweating may limit the extent to which measurements from this site represent whole-body sweat loss [19]. According to the manufacturer, the hDrop is a sweat-based sensor that measures electrical resistance and converts the resulting signal into estimates of sweat composition and hydration-related outcomes using proprietary algorithms [18]. The device also provides an instantaneous hydration assessment when momentary assessments are needed [19]. Although the hDrop may offer a practical approach to field-based hydration monitoring, published evaluations of the device remain limited and have primarily been conducted under controlled conditions [20].

Therefore, the purpose of this pilot study was to evaluate the validity of the hDrop Gen 2 for estimating sweat loss and hydration status during exercise in Division I collegiate male soccer athletes. Specifically, the study evaluated agreement between hDrop-estimated total sweat loss and criterion sweat loss determined from changes in body mass, fluid intake, and urine output. The study also examined the association between the hDrop instantaneous hydration score and urine specific gravity. It was hypothesized that hDrop-estimated sweat loss would be associated with criterion sweat loss and that the instantaneous hydration score would be associated with urine specific gravity.

## 2. Materials and Methods

### 2.1. Study Design and Participants

This pilot study was a field-based method-comparison involving one or more training session observations per athlete to evaluate the validity of a wearable hydration monitor during collegiate soccer training. Participants were male Division I collegiate soccer athletes aged 18 years or older who were members of the University of North Florida, Jacksonville, FL, USA men’s soccer team. Athletes were eligible to participate if they were cleared to participate in their collegiate sport and did not report any contraindications to exercise on the Physical Activity Readiness Questionnaire for Everyone (PAR-Q+).

Participants were recruited from the men’s soccer team and they completed the study procedures during regularly scheduled team practices at the University’s soccer stadium located in north-east Florida. Data were collected during the months of October and November in year 2025, which are during the primary soccer season for Division 1 men’s collegiate soccer. Twenty-four athletes provided informed consent. Of these, twenty-two unique athletes contributed a total of 38 training-session observations. Because some athletes completed the protocol during more than one training session, the number of observations exceeded the number of unique participants.

The study was approved by the University of North Florida Institutional Review Board (IRB-FY2025-88). All participants provided written informed consent before completing any study procedures.

### 2.2. Study Procedures

Participants completed the study protocol during a regularly scheduled soccer training session. Before practice, researchers prepared a private restroom stall for each participant with a 900 mL urine collection hat. Participants were instructed to completely empty their bladder into the collection hat, after which the volume of urine was measured using a 100 mL graduated cylinder, a clinical refractometer was used to measure urine specific gravity (USG), and a 50 mL sample was retained for future analysis. Participants then entered a separate private stall and completed a nude body mass measurement using a digital scale. Immediately after weighing, participants reported the displayed body mass to a researcher, who recorded the value.

Participants then completed an instantaneous hydration assessment using the hDrop Gen 2 Hydration Sensor (hDrop Technologies Inc., Little Rock, AR, USA). Before each data-collection session, the hDrop monitors were charged and connected to the companion application. No separate calibration procedure was performed by the research team. For this assessment, participants placed their thumb on the sensor and remained still while the device assessed hydration status and a hydration score was provided via the hDrop companion app (version 0.50.1) for iPhone. After completion of the hydration assessment, the sensor was snugly secured to the participant’s upper arm with a Velcro strap no more than 5 min before the start of practice.

Each participant was provided with two water bottles containing a known volume of fluid, one containing water and the other containing Gatorade. Participants were permitted to consume both fluids ad libitum during practice. When additional fluid was required, study personnel refilled the bottle with a measured volume. The volume of fluid remaining after practice was measured, and total fluid intake was calculated as the volume provided minus the volume remaining. Any urine produced during the training session was collected and measured.

Participants completed their normal team training while wearing the hDrop sensor. All training sessions were completed outside on a grass or turf soccer field. Participants followed the training activities prescribed by the coaching staff and exercise intensity was not standardized by the research team. All participants completed the full training session which were approximately 90 min in duration. Environmental conditions varied across training sessions, with recorded temperatures ranging from 13.9 to 25.5 °C and relative humidity ranging from 54% to 95%. The device-generated estimate of total sweat loss was obtained from the hDrop companion application following the training session. Participant-level measures of exercise intensity or workload, such as heart rate, GPS-derived metrics, or session rating of perceived exertion, were not available to the research team for these observations.

Immediately after practice, participants completed a second nude body mass measurement in the same private setting. The hDrop sensor was then removed, and participants completed a second instantaneous hydration assessment using the same procedures as the pre-practice assessment. A final urine sample was subsequently collected and using the procedures listed earlier, volume and USG were measured, and a 50 mL sample was stored. Participants were also asked whether they had experienced vomiting or defecation during practice that could have affected body mass change.

### 2.3. Outcome Measures and Calculations

Criterion Sweat Loss. Criterion sweat loss was estimated from changes in body mass corrected for fluid intake and urine output during the training session. Sweat loss was calculated using the following equation:

Criterion sweat loss (mL) = ([pre-practice body mass (kg) − post-practice body mass (kg)] × 1000) + fluid intake (mL) − urine output (mL).

For this calculation, a change of 1 kg in body mass was considered equivalent to 1000 mL of fluid. Fluid intake represented the total volume of water and sports drink consumed during practice. Urine output represented any urine produced between the pre- and post-practice body mass measurements.

hDrop Sweat-Loss Estimate. The hDrop Gen 2 Hydration Sensor was worn throughout the training session and generated an estimate of total sweat loss. The device-derived sweat-loss estimate was obtained from the hDrop companion application following practice and recorded in milliliters. The hDrop estimate was compared with criterion sweat loss to evaluate agreement and individual measurement error.

Instantaneous Hydration Score. The hDrop instantaneous hydration assessment generated a numerical hydration score before and after practice. The score ranged from 0 to 100, with higher scores indicating better estimated hydration status. Scores were obtained from the hDrop companion application and recorded by the research team. Pre- and post-practice hydration scores were evaluated in relation to USG measured at the corresponding time point. Changes in hydration score from pre- to post-practice were also examined.

Urine Specific Gravity. USG was measured from urine samples collected immediately before and after practice using a clinical refractometer. Pre-practice USG was compared with the pre-practice hDrop hydration score, and post-practice USG was compared with the post-practice hDrop hydration score.

Device Output Availability. Following each training session, the hDrop companion application was reviewed to determine whether a sweat-loss estimate had been generated. An observation was considered complete for the sweat-loss validity analysis only when both criterion sweat loss and an hDrop-generated sweat-loss estimate were available. For several observations, no sweat-loss estimate appeared in the application. The reasons for these missing device outputs were unknown. Device-output availability was subsequently evaluated as a binary outcome to examine whether selected session-level factors were associated with the likelihood of obtaining an hDrop sweat-loss estimate.

### 2.4. Statistical Analysis

Descriptive statistics were calculated for all study variables and are reported as mean ± standard deviation unless otherwise indicated. Analyses were conducted using IBM SPSS Statistics, version 29.0 (IBM Corp., Armonk, NY, USA), with statistical significance set at *p* < 0.05.

For the sweat-loss validity analysis, criterion sweat loss and hDrop-estimated sweat loss were compared using mean bias, percent of criterion, mean absolute percent error (MAPE), repeated-measures Bland–Altman analysis, and generalized estimating equations (GEE). Bias was calculated as the hDrop estimate minus criterion sweat loss. Because some athletes contributed more than one observation, repeated observations were accounted for in the inferential and agreement analyses. For the repeated-measures Bland–Altman analysis, the difference between hDrop-estimated and criterion sweat loss was modeled as a function of the mean of the two methods using a linear mixed model with participant included as a random effect. The slope of the mean value was used to evaluate proportional bias. Because significant proportional bias was present, regression-based limits of agreement were used rather than a single fixed pair of limits across the measurement range.

Device-output availability was coded as a binary outcome and examined using binary GEE models accounting for repeated observations within participants. Separate exploratory models evaluated whether criterion sweat loss, body-mass loss, mean session temperature, mean relative humidity, and total fluid consumed were associated with the likelihood that the hDrop generated a sweat-loss estimate.

Percent of criterion was calculated as:

Percent of criterion (%) = (hDrop-estimated sweat loss ÷ criterion sweat loss) × 100.

Absolute percent error was calculated for each observation as:

Absolute percent error (%) = [|hDrop-estimated sweat loss − criterion sweat loss| ÷ criterion sweat loss] × 100.

MAPE was calculated as the mean of the individual absolute percent errors.

A generalized estimating equation with a normal distribution and identity link was used to examine the association between criterion-measured sweat loss and hDrop-estimated sweat loss while accounting for repeated observations within participants. Participant was specified as the subject effect, and an exchangeable working correlation structure was used. Separate generalized estimating equation models with a normal distribution and identity link were used to examine associations between hDrop instantaneous hydration scores and USG before and after practice while accounting for repeated observations within participants. Participant was specified as the subject effect, and an exchangeable working correlation structure was used. Pre- to post-practice changes in hDrop hydration score and USG were evaluated using separate generalized estimating equation models of session-level change scores, with participant specified as the subject effect to account for repeated training-session observations within athletes.

## 3. Results

Twenty-two male Division I collegiate soccer athletes contributed a total of 38 training-session observations (Table 1). Because some athletes completed the protocol during more than one training session, analyses were conducted using training-session observations, and the number of observations exceeded the number of unique athletes.

Complete criterion and hDrop sweat-loss data were available for 18 observations contributed by 16 athletes, representing 47.4% of all training-session observations. Twenty observations were excluded from the sweat-loss validity analyses because the hDrop application did not provide a sweat-loss estimate.

In exploratory binary GEE analyses, greater criterion sweat loss was associated with a higher likelihood of obtaining an hDrop sweat-loss estimate (OR = 2.082 per L, 95% CI 1.302–3.329, *p* = 0.002). Device-output availability was not significantly associated with body-mass loss (OR = 1.188, 95% CI 0.682–2.071, *p* = 0.543), mean session temperature (OR = 0.796, 95% CI 0.542–1.169, *p* = 0.245), mean relative humidity (OR = 0.968, 95% CI 0.908–1.031, *p* = 0.312), or total fluid consumed (OR = 1.025 per oz, 95% CI 0.997–1.053, *p* = 0.086).

For the hydration-status analyses, 36 observations had complete pre-practice hDrop hydration-score and USG data, and 36 had complete post-practice hydration-score and USG data. Complete paired pre- and post-practice hDrop hydration scores were available for 36 observations, whereas complete paired pre- and post-practice USG measurements were available for 37 observations.

### 3.1. Sweat-Loss Agreement and Individual Error

**Figure 1:**
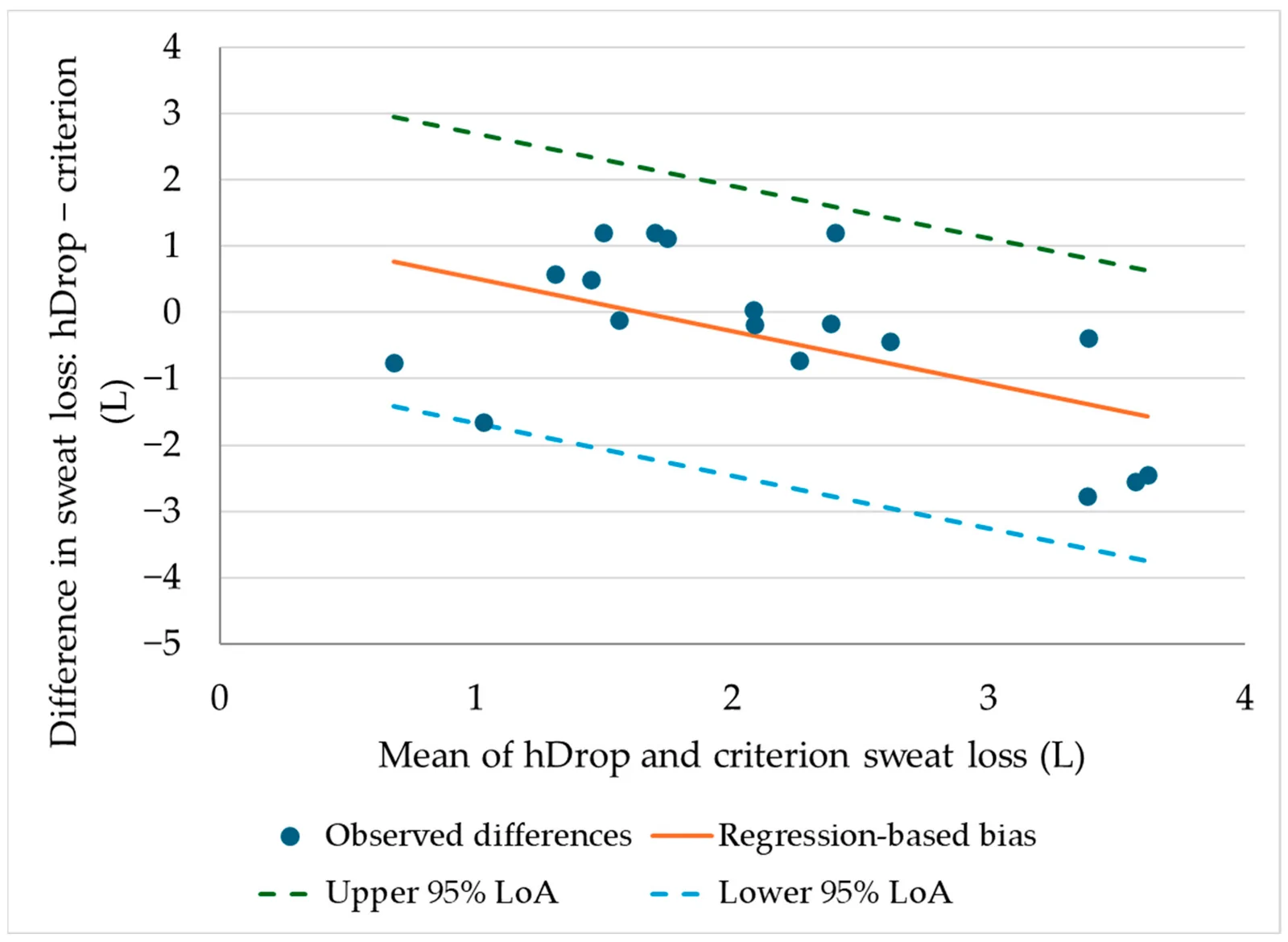
Repeated-measures Bland–Altman plot comparing hDrop-estimated and criterion-measured sweat loss (18 observations from 16 athletes). Differences were calculated as hDrop minus criterion sweat loss; negative values indicate underestimation by the hDrop. The central regression line represents predicted bias across mean sweat-loss values, and the upper and lower lines represent regression-based 95% limits of agreement.

Among the 18 observations with complete sweat-loss data, the repeated-measures mean bias between hDrop-estimated and criterion sweat loss was −0.423 L (95% CI −1.106 to 0.259 L). Significant proportional bias was present (slope = −0.790, 95% CI −1.438 to −0.143, *p* = 0.020), indicating greater underestimation as mean sweat loss increased. Because proportional bias was present, regression-based limits of agreement were used and varied across the range of sweat-loss values (Figure 1). The mean hDrop estimate was 106.2 ± 64.8% of criterion sweat loss, and mean absolute percent error was 50.3%. In the repeated-measures GEE model, criterion-measured sweat loss was positively associated with hDrop-estimated sweat loss (B = 0.204, 95% CI 0.018–0.391, *p* = 0.032).

### 3.2. Instantaneous Hydration Score

Mean hDrop instantaneous hydration scores decreased from 82.3 ± 13.3 before practice to 59.8 ± 20.4 after practice. In a repeated measures GEE analysis, the estimated pre- to post-practice change in hDrop hydration score was −21.97 points (95% CI −30.06 to −13.88, *p* < 0.001).

In repeated-measures GEE analyses, pre-practice USG was not significantly associated with the hDrop hydration score (B = −407.9, 95% CI −1008.7 to 192.8, *p* = 0.183, *n* = 36). Post-practice USG was also not significantly associated with the hDrop hydration score (B = −135.0, 95% CI −803.8 to 533.7, *p* = 0.692, *n* = 36).

### 3.3. Urine Specific Gravity

Mean urine specific gravity decreased from 1.031 ± 0.009 before practice to 1.028 ± 0.010 after practice. In a repeated-measures GEE analysis, the estimated pre- to post-practice change in USG was −0.003 (95% CI −0.006 to −0.001, *p* = 0.009).

## 4. Discussion

In this pilot study, we evaluated the group-level agreement and individual-level accuracy of sweat-loss estimates from the hDrop, a commercially available wearable hydration sensor, and examined the relationship between hDrop hydration scores and urine-specific gravity (USG). The hDrop generated a sweat-loss estimate for only 18 of 38 training-session observations (47.4%). Among observations for which an estimate was available, the repeated-measures mean bias was −0.423 L (95% CI −1.106 to 0.259 L), while mean absolute percent error was 50.3%. Significant proportional bias was also present, with greater underestimation as mean sweat loss increased. Criterion-measured sweat loss was positively associated with hDrop-estimated sweat loss in the repeated-measures GEE analysis; however, association between methods does not itself indicate acceptable agreement. Hydration scores decreased significantly from pre- to post-practice, indicating that the hDrop-generated score changed across the training session; however, these scores were not significantly associated with concurrent USG values. Collectively, the findings indicate substantial limitations in device-output availability and individual-level agreement despite a positive association between hDrop-estimated and criterion-measured sweat loss, along with no meaningful association between the instantaneous hydration score and urine-based hydration status.

Although the repeated-measures mean bias was relatively small, the wide individual error reflected by the 50.3% MAPE and the presence of significant proportional bias indicate that average bias alone should not be interpreted as evidence of acceptable agreement. The positive repeated-measures association between criterion and hDrop-estimated sweat loss indicates that the two measures changed in the same general direction, but does not establish that the methods are interchangeable for individual assessment. In contrast, in a conference abstract, Sellner and colleagues completed a laboratory-based study of hDrop sweat loss estimates using a similar criterion measure of sweat loss (nude body mass pre- and post-exercise) [21]. They found that after 60 min of aerobic activity in a controlled environmental chamber, the hDrop sweat losses were not significantly different from the criterion measure. They, too, demonstrated a relatively small mean bias between assessment methods, however, their study also yielded much narrower limits of agreement. Differences in testing environment and exercise type may have contributed to the differing findings between studies; however, the present study was designed specifically to evaluate device performance under routine field conditions, and these factors should not be considered sufficient explanations for the observed measurement error. Results of both studies highlight the importance of criterion assessment at the group and individual level, specifically when investigating a metric, like exercise sweat rates, that greatly differs among individuals.

Individual estimates of total body sweat loss were highly variable in this study, with an MAPE of 50.3%. This level of error could reflect a combination of physiological and technological factors. One important consideration is that sweat rate varies across different regions of the body. Baker and colleagues measured sweat rate at nine body sites while also independently assessing whole-body sweat loss [22]. They found clear differences among regional sites and showed that a single regional measurement does not perfectly represent whole-body sweat loss. Similarly, Smith and Havenith reported that upper-body regional sweat patterns changed following heat acclimation, which may be relevant because the soccer athletes in the present study were likely accustomed to training in hot conditions [23]. Both Baker et al. and Smith and Havenith also showed that sweat rates are generally greater across the torso than the limbs [19,23].

The hDrop is worn on the upper arm, an accessible location that allows continuous skin contact during exercise. However, because the device relies on a localized skin-based signal from the upper arm to estimate whole-body sweat loss, regional variation in sweating may contribute to error in ways that are not yet understood [20]. Regional sweat rate was not measured in the present study; therefore, its contribution to the observed error cannot be determined from these data. Additional technical factors may also influence wearable sweat measurements. Gao and colleagues noted that flexible sweat sensors can be affected by the mixing of older and newly secreted sweat, contamination from substances on the skin, rapid sweat evaporation, and poor skin contact [24]. Although these challenges were not evaluated specifically for the hDrop, they illustrate the broader difficulty of obtaining consistent measurements from a skin-mounted sensor during exercise. Because technical performance factors such as sensor displacement, signal stability, and device reliability were not directly assessed in the present study, the extent to which these factors contributed to the observed variability remains unknown.

Environmental conditions may further affect both the physiological sweating response and the skin–sensor interface. In the present study, it is unknown whether variation in temperature, humidity, movement, sensor contact, or sweat accumulation contributed to the observed error. In exploratory analyses, neither mean session temperature nor relative humidity was significantly associated with whether the device generated a sweat-loss estimate. However, these analyses addressed output availability rather than measurement accuracy and do not exclude potential environmental effects on sensor performance. Additional research is needed to determine how these physiological, environmental, and technological factors influence hDrop estimates of whole-body sweat loss. Exercise intensity was also not standardized, and participant-level workload measures such as heart rate, GPS-derived metrics, or session-RPE were unavailable; therefore, the potential contribution of exercise intensity to between-session variability could not be evaluated.

In addition to estimating sweat loss, the hDrop provides an instantaneous hydration score. A rapid, noninvasive estimate of hydration status could have practical applications in sport, occupational settings, and daily life across a range of populations. In contrast, urine specific gravity (USG), which is commonly used as a practical indicator of hydration status, requires collection of a urine sample and may be less convenient for repeated or immediate assessment. The hDrop score is obtained through an approximately 80 s measurement during which the user places a thumb on the sensor. To our knowledge, no previous studies have evaluated the validity of the hDrop instantaneous hydration score.

In the present study, the hDrop hydration score decreased significantly from pre- to post-practice, suggesting that the device-generated score was responsive to the practice session. However, USG also decreased, indicating that the urine became slightly more dilute after practice. Thus, although both measures decreased numerically, their physiological interpretations differed: the lower hDrop score was interpreted, according to the manufacturer, as poorer hydration status, whereas the lower USG suggested more dilute urine. Furthermore, hDrop hydration scores were not significantly associated with USG in the repeated-measures analyses either before or after practice.

The lack of agreement between the two measures may reflect differences in the physiological processes and timeframes they represent. USG reflects urine concentration and is influenced by preceding fluid intake and renal regulation of water excretion. Pre-practice USG values were also relatively high and showed a limited range, which may have reduced the ability to detect an association with the hDrop hydration score. Therefore, a urine sample can capture a picture of total body fluid regulation that preceded urine collection rather than acute changes in whole-body hydration at the moment of measurement [25]. In contrast, the hDrop instantaneous score may be more responsive to recent skin- or sweat-related conditions similar to those mentioned the assessment of total sweat loss. Consequently, when these types of measurements are obtained at the same time, they may not respond to exercise-related fluid loss over the same timeframe. Fluid consumed during practice may alter postexercise urine concentration independently of the athlete’s net body–water balance, while exercise-related physiological changes may occur before they are fully reflected in a urine sample. These temporal and physiological differences may help explain the weak associations between the hDrop score and USG. Further research is needed to establish the physiological basis, reliability, and criterion validity of the hDrop instantaneous hydration score.

An additional practical concern was the device-output completion rate. The hDrop generated a sweat-loss estimate in only 47.4% of attempted training-session observations. Moreover, greater criterion sweat loss was associated with a higher likelihood of obtaining an output, suggesting that sessions with available hDrop estimates may not represent a random subset of all attempted measurements. Accordingly, the accuracy findings should be interpreted as applying only to sessions in which the device successfully generated an estimate.

### 4.1. Practical Implications

Considerable variability in sweat loss was observed among athletes, supporting previous evidence that fluid requirements differ substantially between individuals. Accurate assessment of sweat loss therefore remains important for developing personalized hydration strategies aimed at limiting excessive exercise-induced body–water deficits during training and competition. Although the hDrop was not sufficiently accurate for estimating individual sweat loss or hydration status in this pilot sample, wearable hydration technologies may offer practical advantages in athletic settings where traditional methods, such as repeated body-mass measurements, fluid-intake tracking, and urine collection, are difficult to implement routinely.

The significant decline in hydration scores following training suggests that the device-generated score changed across the exercise session; however, because the score was not associated with concurrent USG, the physiological meaning of this change remains uncertain. Although the device provides rapid feedback, the present findings do not establish that the instantaneous hydration score accurately reflects whole-body hydration status; therefore, its practical value for guiding hydration decisions remains uncertain. However, given the substantial individual-level error observed and limited device-output availability observed in this study, the hDrop should not presently be used as a replacement for established methods when precise sweat-loss estimation or individualized hydration recommendations are required.

### 4.2. Strengths and Limitations

A major strength of this study was the field-based evaluation conducted during routine Division I collegiate soccer practices. This approach increases ecological validity because the device was tested under the same conditions in which it would typically be used. Additionally, the monitor was evaluated in a competitive athletic population for whom individualized hydration assessment is often a practical concern. The study also compared hDrop estimates with established field-based measures of sweat loss and hydration status, including body-mass-derived sweat-loss estimates and USG. Furthermore, the assessment of both group-level agreement and individual-level error provided a more comprehensive evaluation of device performance than reliance on mean bias alone. The inclusion of pre- and post-practice hydration measurements also allowed evaluation of changes in the device-generated hydration score across exercise. This study also has several limitations. As a pilot study, the sample size was relatively small, which may have limited statistical power and reduced generalizability. Environmental conditions and factors influencing sweat production were not systematically evaluated as determinants of measurement accuracy and may have affected sensor performance. Exercise intensity was not standardized, and participant-level workload measures such as heart rate, GPS-derived metrics, or session-RPE were unavailable; therefore, their contribution to between-session variability could not be evaluated. In addition, although body-mass change adjusted for fluid balance is a widely accepted field-based approach for estimating sweat loss, it is subject to inherent measurement limitations and may not perfectly reflect true sweat losses. The criterion calculation did not account for respiratory water loss, metabolic mass loss, or sweat retained in clothing, which may have introduced additional error into the field-based sweat-loss estimate. Additionally, USG represents a single-point measure of urine concentration and may not fully capture dynamic changes in whole-body hydration status, which could partially explain the lack of association observed between USG and hDrop hydration scores. A further limitation was the high proportion of sessions for which the hDrop did not generate a sweat-loss estimate. Complete paired sweat-loss data were available for only 18 of 38 observations, resulting in a device-output completion rate of 47.4%. Although the specific reasons for missing device outputs were unknown, greater criterion sweat loss was associated with a higher likelihood of obtaining an hDrop estimate, suggesting that sessions with available data may not represent a random subset of all attempted measurements. The accuracy results therefore apply only to sessions in which the device successfully produced a sweat-loss estimate and may not represent device performance across all training sessions. Inter- and intra-device technical reliability were not assessed because participants did not wear multiple monitors simultaneously and repeated same-time measurements with the same device were not performed. Accordingly, the present study cannot determine the extent to which the observed variability reflects biological variation versus device-related measurement variability. Finally, the study included only male Division I collegiate soccer athletes, limiting generalizability to female athletes, other sports, endurance populations, and recreationally active individuals. Future validation studies should include larger samples, repeated technical reliability testing, independent calibration or technical verification procedures, and statistical methods that appropriately account for repeated observations within participants.

## 5. Conclusions

Taken together, these findings indicate substantial limitations in the field performance of the hDrop in this pilot sample. The device generated sweat-loss estimates in only 47.4% of training-session observations and demonstrated substantial individual-level error and significant proportional bias when estimates were available. Although criterion-measured sweat loss was positively associated with hDrop-estimated sweat loss, the observed agreement does not support use of the device as an interchangeable substitute for established methods of individualized sweat-loss assessment. The physiological meaning and criterion validity of the instantaneous hydration score also remain uncertain. Further refinement and validation are needed before the device can be recommended for individualized hydration assessment in athletes.

## Figures and Tables

**Table 1:** Characteristics of Division I Collegiate Soccer Athletes and Training-Session Observations Included in the Study.

Characteristic	*n*	Mean ± SD	Range
Age (years)	20	21.0 ± 1.6	18–24
Height (cm)	22	182.6 ± 6.1	170.2–193.0
Body mass (kg)	22	78.0 ± 7.9	65.8–94.8
Body mass index (kg/m^2^)	22	23.4 ± 1.9	20.6–29.2

Note: Sample size varies by characteristic because age data were unavailable for two athletes.

## Data Availability

The data presented in this study are not publicly available because they form part of an ongoing study for which data collection and analysis are continuing and because of participant privacy and institutional research-ethics considerations. Requests for access may be directed to the corresponding author and will be considered in accordance with applicable institutional and ethical requirements.
